# Biotransformation profiles from a cohort of chronic fatigue women in response to a hepatic detoxification challenge

**DOI:** 10.1371/journal.pone.0216298

**Published:** 2019-05-10

**Authors:** Elardus Erasmus, Francois E. Steffens, Mari van Reenen, B. Chris Vorster, Carolus J. Reinecke

**Affiliations:** 1 Human Metabolomics, North-West University (Potchefstroom Campus), South Africa; 2 Department of Consumer Science, University of Pretoria, Pretoria, South Africa; Alexandria University, EGYPT

## Abstract

Chronic fatigue, in its various manifestations, frequently co-occur with pain, sleep disturbances and depression and is a non-communicable condition which is rapidly becoming endemic worldwide. However, it is handicapped by a lack of objective definitions and diagnostic measures. This has prompted the World Health Organization to develop an international instrument whose intended purpose is to improve quality of life (QOL), with energy and fatigue as one domain of focus. To complement this objective, the interface between detoxification, the exposome, and xenobiotic-sensing by nuclear receptors that mediate induction of biotransformation-linked genes, is stimulating renewed attention to a rational development of strategies to identify the metabolic profiles in complex multifactorial conditions like fatigue. Here we present results from a seven-year study of a cohort of 576 female patients suffering from low to high levels of chronic fatigue, in which phase I and phase II biotransformation was assessed. The biotransformation profiles used were based on hepatic detoxification challenge tests through oral caffeine, acetaminophen and acetylsalicylic acid ingestion coupled with oxidative stress analyses. The interventions indicated normal phase I but increased phase II glucuronidation and glycination conjugation. Complementarity was indicated between a fatigue scale, medical symptoms and associated energy-related parameters by application of Chi-square Automatic Interaction Detector (CHAID) analysis. The presented study provides a cluster of data from which we propose that multidisciplinary inputs from the combination of a fatigue scale, medical symptoms and biotransformation profiles provide the rationale for the development of a comprehensive laboratory instrument for improved diagnostics and personalized interventions in patients with chronic fatigue with a view to improving their QOL.

## Introduction

Chronic fatigue is a condition commonly reported by patients in primary care practice, whose medical practitioners use diverse definitions and diagnostic labels to describe their symptoms [1]. The complaints expressed about fatigue in stressful and competitive societies range from the condition being unexplained at the one end of the fatigue spectrum [2] but commonly co-occurring with pain, sleep disturbances and depression. This cluster of symptoms associated with fatigue is recognized in functional somatic syndromes such as fibromyalgia (FS) [3] and irritable bowel syndrome (IBS) [4]; it is also common to oncology patients and is associated with a significant decline in their functional status and quality of life (QOL) [5]. Chronic fatigue syndrome (CFS) [6] and myalgic encephalomyelitis (ME) [7] occur at the other end of the fatigue spectrum, with the latter condition characterized by consistent excessive fatigue and fatigability. More broadly, fatigue appears to be one of the chronic non-communicable conditions which are rapidly becoming endemic worldwide [8][9][10][11], prompting the World Health Organization (WHO) to established a project on the development of an international quality of life (QOL) assessment instrument[12].

There is no established biological marker for CFS/ME, nor for chronic or periodic fatigue so that the diagnosis is one of exclusion (DOE) [13] after reasonable effort has been taken to eliminate at least some of the many aetiologies where fatigue is a presenting feature. The lack of objective measures of fatigability is a well-founded frustration to clinicians [14]. However, recent metabolomics studies include metabolic profiling of patients suffering from fatigue. Moreover, they have proposed that broad metabolic perturbations prevail in CFS and/or ME [15][16][17][18]. Although the compounds recorded in these studies are not all identical, the major pathways determined to be affected by ME/CFS relate to energy metabolism. These findings are in accordance with the known link between energy deprivation and fatigue [19]. As the energy demands in biotransformation reactions are key features in pharmacological and toxicological research [20][21][22][23], a link between consistent exposure to environmental toxicants and chronic conditions such as fatigue seems evident. Likewise, interest in the interface between detoxification and the impact of the exposome in health and disease is growing [24][25][26]. Moreover, xenobiotic-sensing nuclear receptors that mediate induction of biotransformation-linked cytochrome P450 (CYP450) genes are emerging as new regulators of the hepatic energy metabolism that connects sensing of the chemical environment, detoxification of xenobiotics and metabolic health [26][27]. These studies unequivocally established that the human genome harbours an extensive number of genes encoding enzymes that primarily metabolize endogenous and exogenous substances. Extensive genetic and functional variation in these genes has complex consequences. Such variability affect enzyme expression or structure and the toxicological level of induced or produced metabolites, the clinical manifestation in the patients and eventually also diagnostic and predictive strategies towards fatigue, as will be discussed below. Apart from the molecular research, these developments therefore also call for renewed attention to a rational basis for alternative strategies to identify the metabolic differences that contribute to health and disease in complex multifactorial conditions like fatigue.

A causal relationship between endogenous or exogenous stimuli, biotransformation responses and fatigue is not unequivocally established. The need for further basic research remains on the role of receptors, transcription factors and signalling cascades in the coordinated regulation of phase I and phase II metabolism, in addition to phase III transport of endogenous as well as of exogenous agents, for modelling of diseases and toxicities induced by xenobiotics. Furthermore, these developments also call for renewed attention to a rational basis for alternative strategies to identify the metabolic differences that contribute to health and disease in complex multifactorial conditions like fatigue. Against this background, this paper addresses chronic fatigue with the focus on the role of biotransformation as an essential metabolic process for maintaining energy homeostasis in patients suffering from the condition. The results presented here are the outcomes of a seven-year study and data collection from 576 female patients suffering from low to high levels of fatigue. They were referred by clinicians to our research unit for assessment of their biochemical profiles of phase I and phase II biotransformation. From assessments of this cohort of patients, we indicate a complementarity between a fatigue scale, medical symptoms and their biotransformation profiles and associated energy-related parameters [28][29][30].

In health research, the concept of perturbation of homeostasis through an experimental challenge to quantify health related processes is well established [31][32][33][34]. The response following a challenge reveals multiple aspects of metabolic health that would not be apparent from studying homeostatic parameters. A perturbation from the homeostatic state formed the conceptual basis of our approach and the laboratory instrument used for measuring the biotransformation profile was based on hepatic detoxification challenge tests through oral caffeine, acetaminophen and acetylsalicylic acid ingestion coupled with oxidative stress analyses [35]. Taken together, the interventions with the xenobiotic substances test for the phase I and four phase II hepatic detoxification pathways and provided the scientific rationale for our proposed biotransformation assessment method. In combination with the multidisciplinary contributions from the clinicians, we propose that a novel combination of the three assessment instruments (a fatigue scale, medical symptoms, and biotransformation profiles) provides foundational knowledge for further development of a comprehensive laboratory instrument for directed personal interventions that we believe can lead to improved QOL in patients across a spectrum of chronic fatigue manifestations.

## Materials and methods

### Ethics statement

This study complied with all institutional guidelines of the North-West University as stipulated by the South African Guidelines for Good Clinical Practice Ethical Guidelines for Research, as well as the terms of the Declaration of Helsinki of 1975 (as revised in 2013) for investigation of human participants. Ethical approval was obtained from the Health Research Ethics Committee (HREC) of the North-West University, South Africa (NWU-00102-12-A1). All written consent was obtained based upon informed decision from all participants. An example of the informed consent form can be found in the S1 File under informed consent.

### Patient procedures and selection

This study involved a prospective, observational and measurement design. Women visiting clinicians with complaints of continuous fatigue of various degrees, and the clinical label given to their fatigue condition (DOE) often reflected both the view of the patient and of the doctor about their state because of the widely varying theories about the potential underlying pathology of chronic fatigue. The clinicians informed the patients about the general intentions of the prospective study and asked whether they would consider participation. Pending informed consent, all cases were potentially eligible for the study, regardless of previous types of treatment. Prior to starting the biochemical assessment measurements, the patients underwent observations and fatigue assessment by the clinicians, and were asked to complete a self-report questionnaire that included a demographic profile and an English version of the revised Piper Fatigue Scale (PFS) [36]. The revised PFS used consisted of 22 numerical items which assessed fatigue experienced by the patients. All items were coded on a 0–10 numeric scale. The revised PFS measured four dimensions of subjective fatigue: behaviour/severity, affective meaning, sensory, and cognitive/mood. To calculate a total fatigue score the cumulative sum of all was divided by the total number of items. In addition, a generally accessible Medical Symptoms Questionnaire (MSQ, included in the S1 File) from the Departments of Medicine, Mercy Hospital and Maine Medical Center, Portland [37] was completed with the assistance of a health consultant for each patient to supplement the information from the PFS. The MSQ contains 19 domains, most with 4 items, totaling 75 indicators to each of which the patient assigns a Likert scale numerical ranking (0 being “never” or “seldom” and 4 being “frequently with severe effect”). Finally, measurement of biochemical parameters from saliva, urine and blood was conducted on samples from all participants who agreed to the investigation.

In total, 673 women with complaints of fatigue were assessed for study eligibility. Of these, 576 were eligible; exclusion criteria for all cases were verified indications of diabetes, chronic high blood pressure, asthma, cancer, arthritis and some other minor clinical conditions, but retaining patients suggestive of fibromyalgia, depression or other affect syndromes. All eligible cases fully completed both questionnaires. The mean age of the participants was 43 years (min.: 18; max.: 82; SD: 14.3), mean body weight 72.0 kg (min.: 40; max.: 150; SD: 17.9) with a mean BMI of 26.3 (min.: 16; max.: 51; SD: 6.3).

### Statistical analysis of the Piper Fatigue Scale

We analysed the data of the modified Piper Fatigue Scale (PFS) as a multidimensional model for fatigue in our patient group (data included in the S1 File). Questions 2–23 (22 questions) of the questionnaire were measured on a scale from 1 to 10. Missing values (meaning that a respondent did not answer a particular question and that was applicable to the data from the selected 576 cases) were replaced by 0. To reduce the dimensionality of the 22 items, factor analysis was performed. The sample size was sufficient to allow for factor extraction and so did not limit the factor possibilities to those outlined in the scoring instructions of the PFS. The PFS as developed for oncology patients measured four dimensions of subjective fatigue: behavioural/severity (6 items: #2–7); affective meaning (5 items: #8–12); sensory (5 items: #13–17); and cognitive/mood (6 items: #18–23) over the 22 items used to calculate the four sub-scale/dimensional scores and the total fatigue scores. Having a clinically different fatigue group, we compared various combinations of extraction methods (Principal Components and Principal Axis Factoring) and rotation methods (Varimax Rotation and Direct Oblimin rotation), all of which indicated that there were two underlying factors in our data set. The first factor was seen to load mainly on items 2–17 and is termed Energy Fatigue, whereas the second factor loads mainly on items 18–23 and is termed Mental Fatigue. The factor sores were calculated as follows: Energy Fatigue score: the mean of items 2–17 (16 items); Mental Fatigue score: the mean of items 18–23 (6 items). The reliability of the measurement for the two factors was evaluated by means of Cronbach’s Alpha and proved to be high (0.974 and 0.971 for the 16 and 6 energy and mental fatigue factors, respectively).

Subsequently, the patients were categorized according to their fatigue scores. The mean Energy Fatigue score for the group was 5.88 (min.: 0; max.: 10; SD 2.59) and 5.21 (min.: 0; max.: 5.21; SD 2.37) for the Mental Fatigue score. Both scores were categorised as follows: Low level fatigue (0 < score < 4); Medium level of fatigue (4 ≤ score < 7) and High level of fatigue (score ≥7). These thresholds are based on the severity codes included in the scoring instructions of the PFS, namely: None (0), Mild (1–3), Moderate (4–6), and Severe (7–10). From these levels we defined five combined groupings: (1) Low Fatigue: Energy Fatigue score and Mental Fatigue score below 4; (2) Medium Fatigue: Energy Fatigue score and Mental Fatigue: score below 7 and at least one of the scores equal to 4 or more; (3) Mainly Energy Fatigue: Energy Fatigue score 7 or more, Mental Fatigue: score below 7; (4) Mainly Mental Fatigue: Mental Fatigue score 7 or more, Energy Fatigue: below 7; (5) Energy and Mental Fatigue: Energy Fatigue score and Mental Fatigue score both 7 or more.

### Statistical analysis of the Medical Symptoms Questionnaire

This questionnaire has 75 questions divided into 15 main sections; one question in the energy section (about hyperactivity) was omitted to enhance the reliability. The scores were calculated as the mean of the items per section and the items were also combined in an overall MSQ score. The reliability per section ranged from 0.582 for the 5 items defining MSQ related to “Skin”, and 0.858 for the 6 items related to “Joint”. For this data set the Factor Analysis Rotation and Extraction methods did not converge, and PCA did not provide useful outputs. We therefore decided to calculate factor scores based on the sections and total.

### Statistical profile of the biochemical data

The data consist of 15 chemical measurements to define the biochemical variables and an additional variable for measurement of the filtrate volume required for some calculations. In addition the complete data set also included five ratios which we considered to be informative as well: (1) total acylcarnitine to free carnitine ratio; (2) uric acid to creatinine; (3) phase I to phase II glucuronidation; (4) phase I to phase II sulphation; and (5) phase I to phase II glycination ratio. Statistical analysis of the biochemical variables and ratios revealed extensive skewness and kurtosis of the original data that could be improved by log_e_-transformation of the original data. In addition to analysis of the data for the individual biochemical variables, an inherent underlying relationship among the measurements was apparent. Since the logarithm of a ratio is equal to the difference between the logarithms, this meant that some of the transformed variables are linear combinations of other variables; thus the covariance matrix was singular and factor analysis was not possible; because the variables represent precise measurements rather than opinions, principal components analysis (PCA) is considered to be a viable option. PCA suggested that 8 factors (designated as BioF1 to BioF8) showed a strong underlying relationship among the biochemical data. These factors constitute the underlying relations among the components of the factors. “Furthermore, the human urine metabolome confirmed variation in urine metabolites, based on the populations (age, gender) from which the concentrations of the metabolites were derived[38]. In our data set, confounders were confirmed if showing a practically significant correlation (|r|≥0.5). While we report on the p-values, the level of significance was not considered as this metric is known to be affected by sample size. A sample size of 576, as used here, is likely to produce statistically significant effects of little practical importance. Taken together, from the correlation analysis of our data it appeared that the age, height, weight or BMI of the present experimental group did not have a confounding effect on the biochemical data or on the classification of fatigue groups. (The correlation analysis with potential confounding effects is shown in Table A in the S1 File).

### Statistical methods used

The analysis of all data generated from the investigation was performed according to well-described and validated methods[39] [40].

### Biotransformation analysis

**Fig 1:**
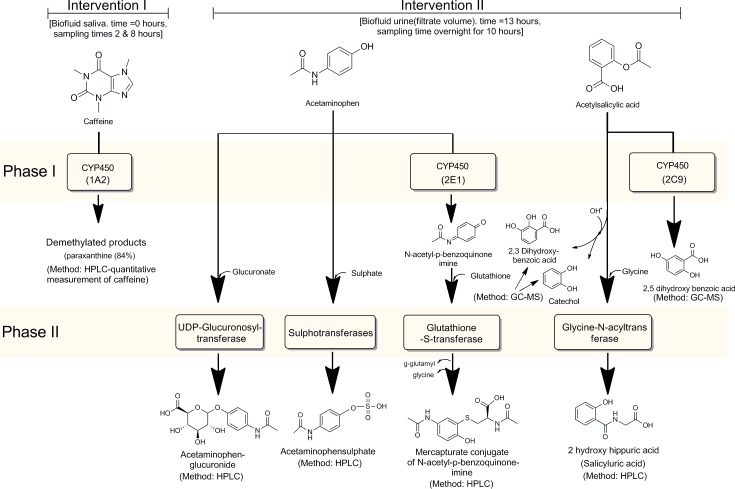
Measurement design for the interventions to assess the biotransformation profiles for all fatigue patients. Caffeine clearance (Intervention 1) progresses through CYP450-1A2, comprises only a phase 1 process and is measured as the residual substrate in saliva, expressed as a percentage of caffeine recovery. The acetaminophen challenge involves three phase II conjugation reactions, evaluated through the products formed following biotransformation: (1) conjugation of glucuronic acid to the hydroxyl group of acetaminophen; (2) sulphation of the phenolic hydroxyl group of acetaminophen; and (3) glutathione conjugation of N-acetyl-p-benzoquinone imine, a phase 1 activated form of acetaminophen. In the simultaneous acetylsalicylic acid challenge, glycination of the functional carboxylic group is catalysed by glycine-N-acyl transferase (GLYAT). All the substances mentioned are measured in urine samples, analysed by HPLC, as indicated. The acetaminophen and salicylate ingestion detoxification are measured through the glucuronide, sulphate and mercapturate conjugates as well as for salicyluric acid, expressed as percentage recovery. Three derivatives of salicylate may likewise be observed in this challenge (2,3-DHBA, 2,5-DHBA and catechol) and are measured by a GC-MS method. Abbreviations: CYP450-1A2, -2E1 and -2CP: mixed function CYP450 oxidase system with isozymes as indicated; GLYAT: glycine-N-acetyltransferase; HPLC: high-pressure liquid chromatography; GC-MS: gas chromatography–mass spectrometry; GSH: glutathione; 2,3-DHBA: 2,3-dihydroxy benzoic acid: 2,5-DHBA: 2,5-dihydroxybenzoic acid.

The measurement design for the biotransformation interventions is shown schematically in Fig 1.

The first intervention is a caffeine clearance test, followed after a fixed period by acetaminophen and acetylsalicylic acid interventions. The biofluids used in the study consisted of saliva, blood and urine. Samples were collected for analysis after challenge substances provided in the test kit were taken by the participants. Phase I biotransformation was challenged with a 150 mg caffeine tablet before breakfast at 8h00 in the morning. Saliva samples were collected in salivettes at 10h00 and 16h00. The phase II biotransformation challenge involved two aspirin (600 mg) and two paracetamol tablets (1000 mg) taken at 21h00 in the evening and overnight and early morning (ending at 7h00) urine samples collected in a special container provided in the test kit. Blood samples (2 EDTA and 1 clotted tube) for additional biochemical analyses were collected 24 hours later when the frozen urine and saliva samples were handed in at a pathology depot for selective analyses as indicated. The methods used for the measurement of phase I and II biotransformation are new and are described below with additional technical information presented in the S1 File.

#### Phase I analysis

Sample preparation: C18 solid phase extraction cartridges (HF Bond Elut C18–100 mg) were used for sample clean-up and extraction of caffeine; 200 μl internal standard 2-acetamidophenol (5 μg/ml) was added to 200 μl saliva sample prior to extraction. Conditioning of the SPE columns was performed with 1 ml methanol and 2 ml water. A 200μl sample with 200μl internal standard was added to the conditioned SPE column and washed with 1 ml water. Samples were eluted with 1 ml methanol acetate solution (pH 3.5), dried under nitrogen and then lyophilized. Samples were reconstituted in 200 μl after which 5 μl was injected into the HPLC. The Agilent 1200 HPLC system was equipped with a binary pump, inline degasser, auto sampler, heated column compartment and diode array detector. The analytical column was an Eclipse XDB C18, 5 μm, 4.6 x 150 mm reverse phase column. A flow of 1 ml/min was used, column temperature 25°C and the UV detector set at 273 nm. From a caffeine stock solution (1 mg/100 ml), a six-point series dilution calibration range was made up ranging from 0.156 μg/ml to 5 μg/ml for quantification.

#### Phase II analysis

Aliquots of 500 μl from the 10 hour overnight collected samples were centrifuged and 200 μl of each sample combined with 200 μl internal standard (Allopurinol 250 μg/ml) for the HPLC analysis. The analytical column was a Phenomenex Luna 3μ C18 (2) 100 A, 150 x 2.00 mm reverse phase column. Column temperature was 35°C, flow rate 0.25 ml/min and the UV detector was set at 254 nm for analyte detection. Mobile phase A was H_2_O with 0.05% TFA and mobile phase B was HPLC-grade methanol. Chromatographic separation of analytes was achieved with the following gradient mobile phase composition: 10% B from 0–2 min; an increase to 20% B to 16 min, from 16–19 min to 90% B and from 19–24 min 90% A and 10% B. To accommodate physiological concentrations for the different conjugates stock solutions a 9-point series dilution calibration range were used for each conjugate, ranging from 3.905 μg/ml to 1000 μg/ml.

#### Secondary products from the acetylsalicylic acid challenge

The three secondary products from the acetylsalicylic acid challenge–catechol, 2,3-DHBA and 2,5-DHBA–were analysed using GC-MS. Creatinine-corrected urine volumes were subjected to organic solvent extraction after 3-phenyl butyric acid was added as internal standard and the pH adjusted to 1 with 5N HCl. Six ml HPLC grade ethyl acetate and 3 ml diethyl ether were used as extraction solvents. After roto-torque agitation for 10 minutes, the solvent layer was removed and dried under a stream of nitrogen. Derivatization was done with BSTFA and TMCS. An Agilent 7890A gas chromatograph connected to an Agilent 5975B MSD mass spectrometer was used for analysis. The analytical column used was a 30 m x 0.25 mm x 0.25 mm DB1-MS JW Scientific column. High purity helium was used as carrier gas. A 1 μl sample was injected with a temperature programme starting at 100°C for 2 min, increased by 4°C to 120°C, then by 6°C to 215°C and finally by 20°C to 300°C. Post-run time was 1 min at 295°C. Selective ion monitoring was conducted for catechol (254.1 m/z), IS (221.1 m/z), 2,3-DHBA (355.2 m/z) and 2,5-DHBA (355.2 m/z). An 8-point calibration range of the standards was used for final quantification.

#### Ferric reducing antioxidant assay (FRAP assay)

Measurement of the ferric reducing ability of plasma, known as the FRAP assay, provides a way for assessing ‘antioxidant power’ and which we used in the present study. FRAP assays were performed on a BIO-TEK FL600 fluoresence microplate reader. Amounts of 15 μl serum were added to 85 μl Milli-Q water in 96-well microplates. The sample plates were incubated for 10 min at 25°C. To this, 250 μl FRAP reagent (2.5 ml FRAP acetate buffer; 2.5 ml TPTZ reagent and 2.5 ml FeCl_3_ reagent) was added. FRAP acetate buffer: 1.55 g anhydrous sodium acetate in 500 ml Milli-Q water. TPTZ reagent: 312.4 mg 2,4,6-tris-2-(pyridyl)-1,3,5-triazine in 100 ml 40mM HCl. FeCl_3_ reagent: 4.56 mg of FeCl_3_.6H_2_O was dissolved in 100 ml FRAP acetate buffer (300mM). FRAP values were recorded 80 seconds after addition of the FRAP reagent. A 6-point calibration range containing 0, 20, 40, 60, 80 and100 μl of a FRAP standard (0.275 FeSO_4_.7H_2_O in 100 ml milliQ H_2_O) was used as calibrator.

#### Reactive oxygen species (ROS assay)

ROS assays were also conducted on a BIO-TEK FL600 fluoresence microplate reader; 140 μl sodium acetate (0.1M; pH 4.8) was added to the wells used in the 96-well plate; 2.5 μl samples were added in triplicate; 100 μl of a 1:25 DEPPD (100 mM) and FeSO_4_ (43.7 μM) solution was added to the sample wells. Plates were incubated at room temperature for 1 min; plates were read at 546 nm every minute for 10 min. A calibrator concentration range with 6 concentrations ranging from 0–300 mg/l hydrogen peroxide was used for quantification of samples.

#### Total glutathione (T-GSH) assay

Total glutathione levels in blood samples were determined using the GSH/GSSG kit from Oxis International. Whole blood EDTA vacutainer samples were mixed and 350 μl metaphosphoric acid (MPA) mixed with 50 μl blood sample, centrifuged at 10 000 x g for 10 min; 12.5 μl of the MPA extract was then added to 750 μl assay buffer. A 50 μl sample was then added in triplicate to the microplate and mixed for 5 min; 50 μl NADPH was then added and the plates were read at 412 nm for 3 min at 1-minute intervals. GSH (0–3.0 μM) and GSSG (0–0.15 μM) 6-point calibration ranges were used for quantification.

#### Creatinine and uric acid

Both creatinine and uric acid values in urine were determined using the Konelab T series analyser with the uric acid (AOX) and creatinine (enzymatic) kits.

#### Carnitine and acylcarnitine analysis

Carnitine and acylcarnitine were measured on the 10-hour urine samples. Urine (100 μl) was transferred to an Eppendorf tube and centrifuged at 1300 rpm for 20 min; 10 μl of the centrifuged urine was added to 410 μl of a deuterated carnitine/acylcarnitine stable-isotope solution in a new Eppendorf tube. The sample mixture was then dried under nitrogen at 65°C for ~15 min; 100 μl 3N butanolic HCl was added to the dried sample and incubated at 65°C for another 15 min. Excess butanolic HCl was evaporated under nitrogen. The samples were then reconstituted in 100 μl acetonitrile: water (50:50) (v/v) with 0.1% formic acid. Samples were delivered to the electrospray mass spectrometer (Agilent 6410 Triple Quadrupole, Palo Alto, CA) via an Agilent 1200 series LC (Palo Alto, CA) and 96-well plate sampler. A 10 μl sample was injected per analysis at a constant mobile phase (1% formic acid in water: acetonitrile (50:50) (v/v)), flow rate of 0.2 ml/min. The mass spectrometry analyses were carried out in positive ionization mode with controlled collisional induced decomposition (fragmenter voltage 135 V and collision energy 20 V) and the precursor of 85 Da function used to selectively detect the different carnitine species. The deuterated analogues of the different carnitine and acylcarnitines (working solutions: free carnitine = 1.8815 μmol/l; acetylcarnitine = 0.6075 μmol/l; propionylcarnitine 0.1616 μmol/l; isovaleryl carnitine = 0.1400 μmol/l; octanoyl carnitine = 0.1233 μmol/l and palmitoyl carnitine = 0.3144 μmol/l) were used for the quantification of individual carnitine species. Linear regression calculations were used to determine the concentration of the carnitine/acylcarnitines by comparison of the signal intensity of carnitine/acylcarnitines against that of the corresponding deuterated stable isotopes. Concentrations of the analysed carnitine and acylcarnitines were expressed in mmol/mol creatinine.

#### Reagents

Caffeine, allopurinol, paracetamol-glucuronide, paracetamol sulphate, high-purity trifluoroacetic acid, salicyluric acid, N,N-diethyl-paraphenylenediamine (DEPPD), sodium acetate, metaphosphoric acid (MPA) and hydrogen peroxide were supplied by Sigma Chemicals. Paracetamol-mercapturate was supplied by Toronto Research Chemicals. HPLC grade acetonitrile and water was supplied by Burdick & Jackson. Ferrous sulphate was supplied by Labchem. Acetamidophenol, phenylbutyric acid, 2,3 & 2,5 dihydroxy benzoic acid, NADPH, GSH, GSSG and catechol were all obtained from Sigma-Aldrich. Ethyl acetate and diethyl ether were purchased from Merck. N,O Bis(trimethylsilyl)fluoro acetamide (BSTFA) and trimethylchlorosilane (TMCS) were supplied by Sigma Aldrich. 2,4,6-tris-2-(pyridyl)-1,3,5-triazine (TPTZ) and Ferri Chloride (FeCl_3_.6H_2_O) were also supplied by Sigma Chemicals. Carnitine and acylcarnitine reference standards and deuterated analogues were obtained from Cambridge Isotope Laboratories.

## Results

**Fig 2:**
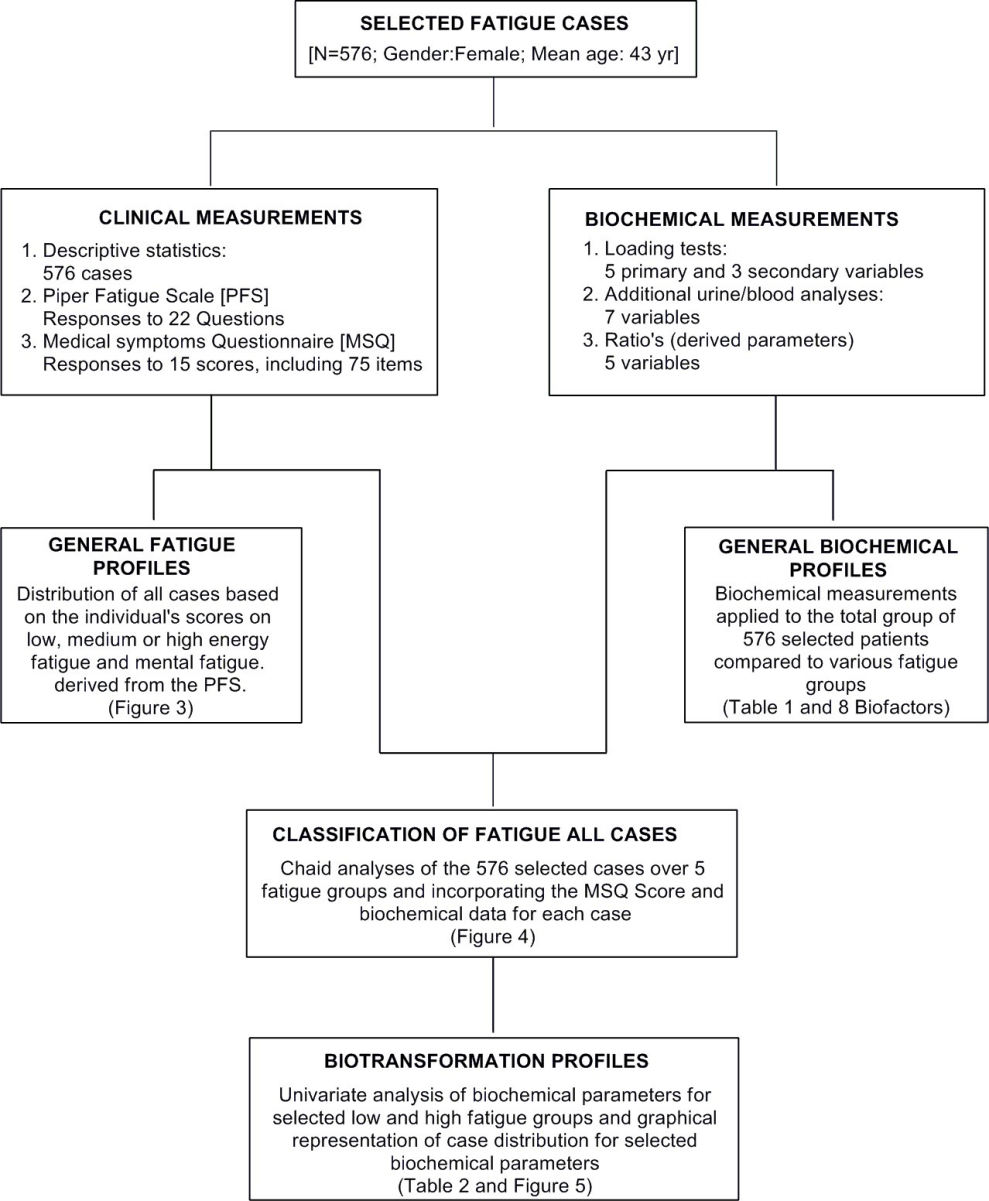
Schematic flow diagram for the analysis of clinical and biochemical data for exploratory biotransformation profiling of samples from all fatigue patients.

A flow diagram of clinical and biochemical data acquisition procedures, comprehensive approaches of the statistical analyses and resulted outcomes is shown schematically in Fig 2.

**Fig 3:**
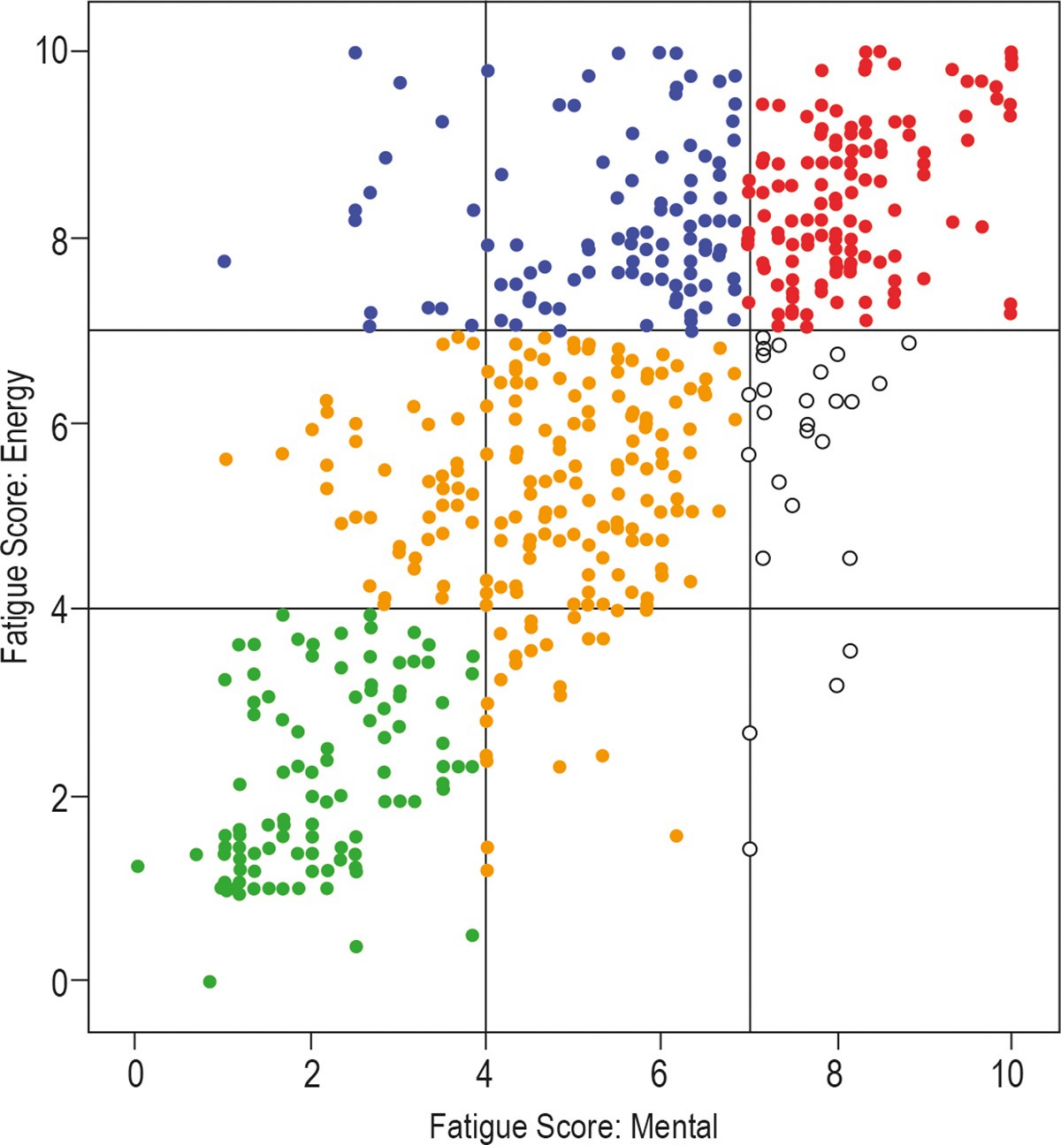
Distribution of all selected cases based on the individual scores in terms of energy fatigue and mental fatigue derived from the PFS. The scores are categorized as low level fatigue (0 < score < 4), medium level of fatigue (4 ≤ score < 7) and high level of fatigue (score ≥ 7). This distribution indicates the presence of five PFS groups: Low fatigue (green), mild fatigue (orange), mainly energy fatigue (blue), mainly mental fatigue (open circles), and high energy and mental fatigue (red).

The five categories of fatigue patients according to the PFS are shown in Fig 3. As reported by the physicians, the level of fatigue experienced by these subjects ranged from low (indicated by 117 blue dots in Fig 3; 20.3% of the group) to high (130 red dots in Fig 3; 22.6%). The analysis of the PFS of the mild, energy only and mental only fatigue categories were 196 (corresponding to 34.0% of the survey population), 107 (18.6%) and 26 (4.5%), respectively. These results clearly indicate that a physical experience of energy depletion, rather than a psychological mood of mental fatigue, dominates in the experimental group. The descriptive information (mean values and standard deviations) on the 15 original biochemical measurements over the five fatigue groups, as defined in Fig 3, are listed in Table 1.

**Table 1:** Descriptive statistics of the biochemical variables and their manifestation across the original data for the five fatigue groups.

Biochemical Measure	Mean (SD)				
	Low Fatigue (N = 117)	Mild Fatigue (N = 196)	High Energy & Mental Fatigue (N = 130)	Mainly Energy Fatigue (N = 107)	Mainly Mental Fatigue (N = 26)
1. Caffeine Clearance Test					
Phase I	0.64	0.66	0.60	0.61	0.56
	(0.59)	(0.53)	(0.53)	(0.53)	(0.45)
2,5-DHBA	28.02	25.51	29.37	31.20	32.94
	(23.68)	(22.62)	(20.56)	(63.41)	(31.1)
2. Acetaminophen Challenge Test					
Glucuronidation	27.89	29.95	30.53	31.22	26.15
	(13.19)	(15.91)	(15.15)	(15.44)	(10.89)
Sulphation	17.35	17.32	17.51	15.82	15.44
	(8.38)	(8.13)	(8.93)	(7.65)	(6.45)
Glutathione Conjugation	1.56	1.73	1.74	2.50	1.48
	(0.86)	(1.02)	(1.27)	(6.09)	(0.82)
3. Acetylsalicylic Acid Challenge Test					
Salicyluric acid	10.48	12.16	11.25	11.39	10.02
	(5.37)	(7.25)	(5.51)	(5.6)	(5.06)
4. Variables Related to Oxidative Stress					
Catechol	13.41	13.75	12.99	13.34	10.31
	(14.37)	(24.14)	(12.12)	(14.43)	(7.98)
2,3-DHBA	4.27	5.17	4.31	3.61	4.24
	(4.82)	(5.59)	(4.42)	(3.02)	(5.26)
FRAP*	351.48	353.47	353.23	332.09	396.78
	(81.35)	(67.96)	(63.68)	(70.66)	(110.53)
GSHt**	860.89	876.88	873.47	846.50	895.68
	(182.95)	(181.19)	(197.39)	(174.23)	(180.09)
ROS***	107.25	109.64	106.88	110.72	103.75
	(33.37)	(39.44)	(34.89)	(39.74)	(38.93)
5. Other					
Uric acid	1.60	1.83	1.55	1.83	1.26
	(1.16)	(1.49)	(1.31)	(1.42)	(0.73)
Creatinine	8.59	10.01	8.81	9.47	9.63
	(6.1)	(6.24)	(6.14)	(6.36)	(6.75)
Total Acylcarnitine	5.36	5.15	4.76	4.55	3.88
	(3.88)	(3.65)	(3.48)	(2.96)	(2.52)
Total Free Carnitine	4.75	4.83	4.43	3.57	3.10
	(3.58)	(4.77)	(4.23)	(2.19)	(2.3)

* FRAP- Ferri reducing ability of plasma

**GSHt- total red blood cell glutathione

***ROS- Reactive Oxygen Species

An explorative comparison of the original data for the low fatigue group with the four other fatigue groups implies some trends based on the mean values, whereas the trends based on the untransformed data from the 576 patients do not prove to be significant (p > 0.05). The main trends observed are: (1) Somewhat lower phase I biotransformation (caffeine clearance) in both the Mainly Energy Fatigue [0.61% (SD = 0.53)] and Mainly Mental Fatigue [0.56% (0.45)] groups relative to the Low Fatigue [0.64% (0.59)] group. (2) This trend also seems to prevail for phase II biotransformation. (3) The antioxidant capacity in the Mainly Mental Fatigue [FRAP = 396 μmol/l (SD = 110); GSHt = 895 μmol/l (180)] group seems to be better than in the Low Fatigue [FRAP = 351 μmol/l (SD = 81); GSHt = 860 μmol/l (182)] group, which is reflected in apparent less oxidative stress in the Mainly Mental Fatigue [ROS = 103 μmol/l (SD = 38); catechol = 10 μmol/l (7) ] than in the Mainly Energy Fatigue [ROS = 110 μmol/l (SD = 39; catechol = 13 μmol/l (SD = 14)] groups.

To explore these trends further, statistical clusters from the data were formed to identify potential associations between the biochemical measures and fatigue scores, for which two strategies were considered: (1) using factor analysis to identify any underlying structure in the biochemical measures observed, and (2) using a CHAID analysis to refine the 5 fatigue groups. The factor analysis of the Z-scores of the log_e_-transformed data suggested that 8 factors showed a strong underlying relationship among the biochemical data and some ratios between the variables, with Biofactor 8 consisting of only the total acylcarnitine/total free carnitine ratio. The approach used for the factor analysis was adopted as the various metabolites were measured on different scales. This analysis indicates the underlying relation between the components of the factors and supports the selection of variables which we used for our biotransformation instrument. The 7 biofactors which consist of more than one indicator, including our broad interpretation, are:

Biofactor 1: (Phase 1; Phase 1/Phase II glucuronidation ratio); Phase 1/Phase II sulphation ratio; Phase I/Phase II glycination ratio). This biofactor refers to phase I activation in relation to the measured phase II conjugation reactions.

Biofactor 2: (Phase II glucuronidation, sulphation, mercapturation and glycination). These four variables indicate a strong underlying relationship between the four phase II conjugation reactions.

Biofactor 3: (Creatinine and uric acid). The mean urinary creatinine and uric acid excretions in the patients with gout were reported to be significantly increased as compared with those of normal controls[40], but this link is unlikely to be associated with fatigue.

Biofactor 4: (Total free carnitine and total acyl carnitine). These two variables are important indicators of fatty acid metabolism and whether enough free carnitine is available to transport long-chain fatty acids across the mitochondrial membrane. They may indicate a redox imbalance.

Biofactor 5: (Catechol, 2,3-DBHA and 2,5-DBHA). These three metabolites are formed following the acetylsalicylic acid challenge, as indicated in Fig 1, with catechol and 2,3-DBHA formed through free radical mechanisms (indications of oxidative stress) and 2,5-DHBA through phase I, catalysed by a different CYP450 isozyme from in the caffeine challenge.

Biofactor 6: (Uric acid and uric acid: creatinine). Creatine phosphate is catabolized to creatinine in ATP depletion, which may cause accelerated urate synthesis.

Biofactor 7: (ROS; FRAP; GSHt). The combination of these three variables indicates an obvious relation between antioxidant capacity (endogenous GSH and an experimental measure of antioxidant capacity through FRAP) and reactive oxygen species (ROS).

Studies on human liver microsomes indicated the formation of both 2,3- and 2,5-DHBA from salicylic acid (Biofactor 5), but apparently only 2,5-DHBA is produced by the microsomal fraction challenged through aspirin. Catechol, the third member of Biofactor 5, is a secondary product likewise resulting from a free radical reaction. A comparison between the clinical and biochemical data indicates that the canonical correlation between two sets of variables from the clinical data was high for the 22 items from the PFS and from the MSQ (0.789 for 15 items and 0.048 for the 75 sub-items), but not so for the correlations with the biochemical factor data [PFS (22 items) and biochemical factors (8 items): 0.299; MSQ (15 scores) and biochemical factors (8 items): 0.598; MSQ (75 sub-items) and biochemical factors (8 items): 0.484].

**Fig 4:**
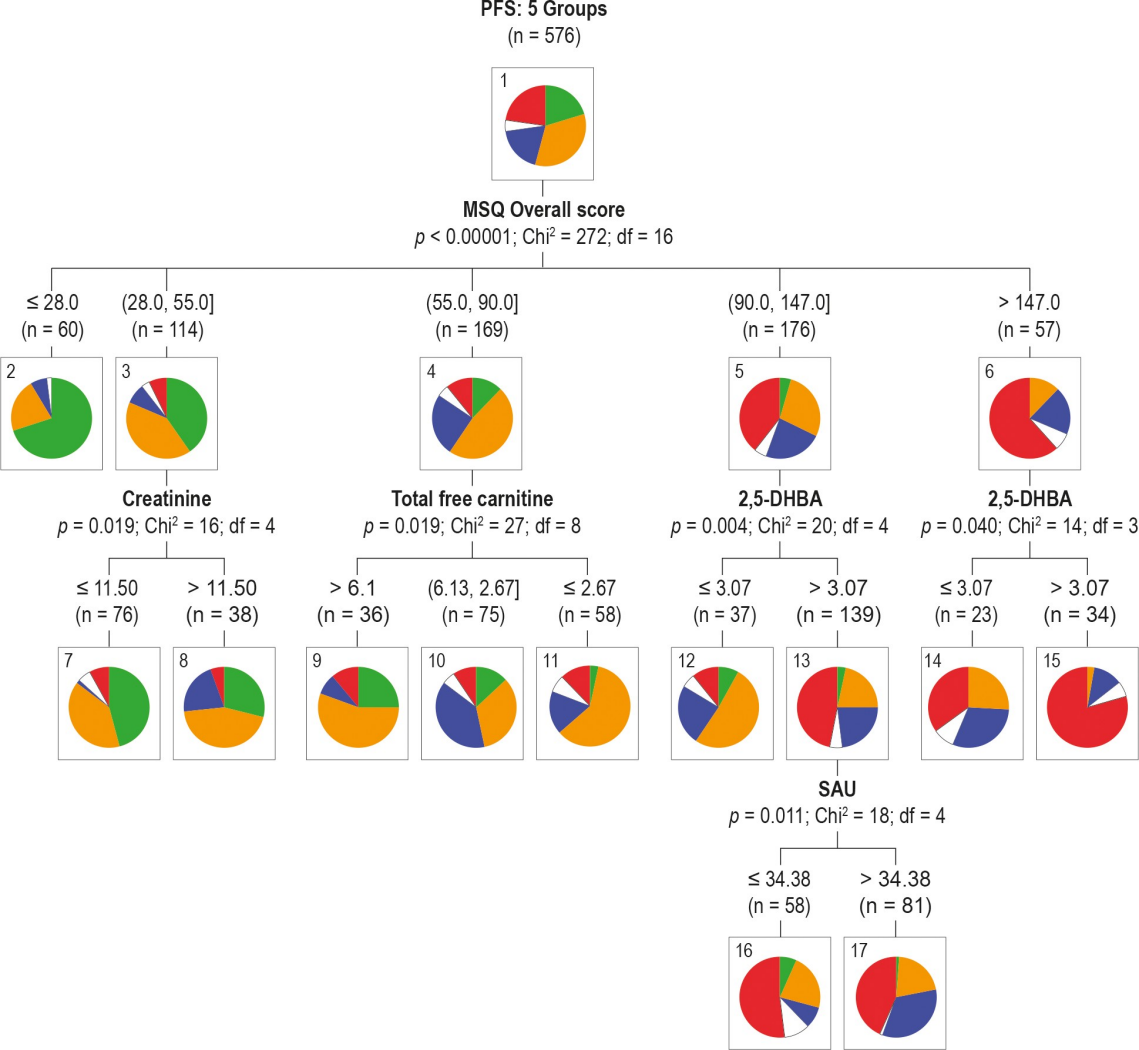
CHAID analysis of the 576 selected cases over 5 fatigue groups (colour code as in Fig 2), incorporating the data from the biochemical and MSQ score analysis.

Subsequently, the data were subjected to a CHAID analysis, used as a classification instrument and for detection of interaction between the PFS grouping, on the one hand, and MSQ and the biochemical variables, on the other hand. CHAID classification is based on Bonferroni-adjusted significance testing and is a type of decision tree. Like other decision trees, CHAID's advantage is that its output is highly visual and easy to interpret. The visual model (Fig 4) clearly highlights how the medical profiles and metabolic features correspond to specific fatigue states.

Node 0 in Fig 4 shows that the highest percentage of the 576 individuals (34%, 196 women) experienced mild fatigue whereas (22.6%, 130) experienced high fatigue. Based on the MSQ overall score, the CHAID analysis next subdivided the individuals into 5 nodes (nodes 1–5). On the two extremes of the MSQ score (< = 28 and 147), 70% of the 60 cases in node 1 experienced low fatigue whereas 61.4% of the 57 cases in node 5 experienced high energy and mental fatigue. The next subdivision produced 9 nodes (nodes 6–14). The biochemical variables assigned to this subdivision were creatinine (in the mild fatigue node 2; MSQ 28–55), total free carnitine (in the mild fatigue node 3; MSQ 55–90) and 2,5-DHBA (in the energy and mental fatigue nodes 4 and 5; MSQ 90–147 and MSQ >147). 2,5-DHBA is of special interest as a discriminator between mild and high energy and mental fatigue. The high fatigued state is associated with levels of 2,5-DHBA >3.067 (node 12). Node 12 is further subdivided into nodes 15 and 16, where 81 of the 139 cases in node 12 displayed glycine conjugation values >34.38.

**Fig 5:**
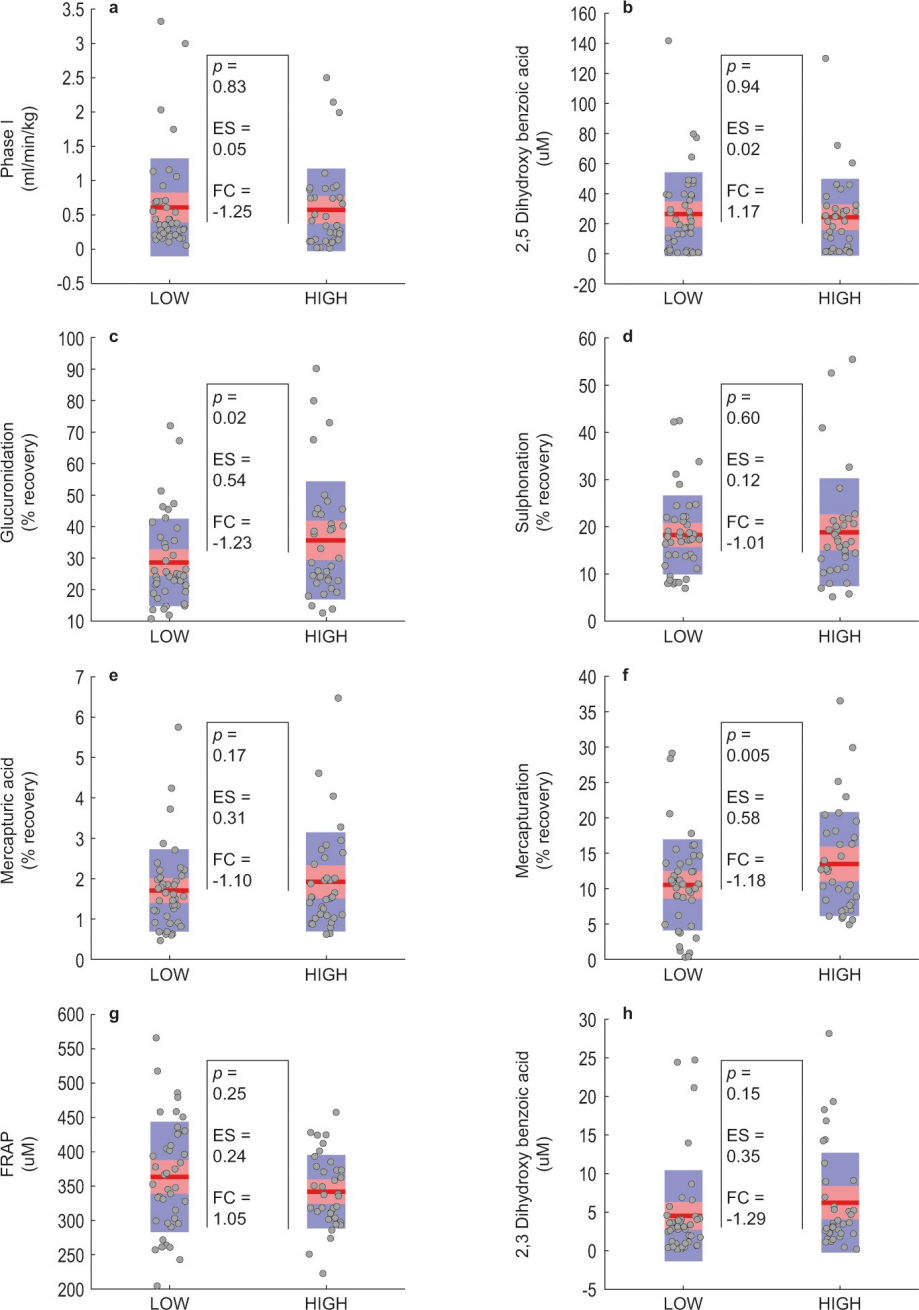
Graphs showing potentially important metabolites for the low and high fatigue groups, selected according to the CHAID classification. Indicated in the figure are: Patients with low fatigue (graphs to the left) relative to those with high fatigue (graphs to the right) in each panel. Values for all individual cases are shown as dots, whereas the squared area represents the 95% confidence interval (orange) and one standard deviation (blue) about the mean (red line). Fold change (FC) measures are indicated for the low relative to the high fatigue groups, and were determined from the original experimental data. The statistical and practical significance of differences (*p*-values and ES) were calculated from the transformed data. The respective p, ES and FC values shown in Fig 5 were calculated as mentioned above, but all data points in these figures represent the original experimental values.

Finally, we investigated the biochemical data of a low and high fatigue group, based on the CHAID classification. The low fatigue group consisted of 37 cases, having PFS <4.0 for energy as well as mental fatigue and a score from the MSQ ≤ 28. The high fatigue group also comprised 37 subjects, having PFS >7.0 for energy as well as mental fatigue and a score from the MSQ >147. We used the original data through a parametric analysis for determination of mean group values and the fold changes (FC), and the transformed data for determination of significance, expressed as p*-*values and effect sizes (ES), shown graphically for important variables in Fig 5.

The graphical data indicate that phase I biotransformation was comparable between the low and high fatigue groups (Fig 5A and 5B). The mean percentage detoxification observed from the three biotransformation modes following the acetoaminophen challenge (27.89:17.53:1.53% recovery and 30.53:17.52:1.74% recovery for glucuronidation:sulphation:mercapturation for the low and high fatigue groups as shown in Table 1), resembles the detoxification profiles reported in the literature (glucuronidation > sulphation > mercapturation) [35]. Moreover, the acetoaminophen challenge clearly indicated that phase II glucuronidation (Fig 5C) was significantly increased in the high fatigue group (FC = +1.23; p *=* 0.02), with practical significance (ES = 0.54). Phase II sulphation did not differ between the low and high fatigue groups (FC = +1.01), but Phase II mercapturation was somewhat increased (FC = +1.10) in the high fatigue group (Fig 5D and 5E, respectively). Salicyluric acid excretion following the salicylic acid challenge was significantly higher (FC = +1.18; p = 0.00) in the high than in the low fatigue groups (Fig 5F), which also appears to be practically significant (ES = 0.58). The statistical metrics shown for FRAP (Fig 5E) and 2,3-DHBA (Fig 5F) resemble the trends observed for these values as shown in Table 1. Taken together, these results support the factor analysis which indicated a strong underlying relationship between phase I and II biotransformation (Biofactor 1), between the four modes of phase II biotransformation (Biofactor 2) as well as on the relation between high antioxidant capacity and low oxidative stress (biofactors 5 and 7).

With regard to the remaining supplementary biochemical measurements, we observed a decrease in the total acylcarnitine within the high fatigue group (FC = –1.25; p = 0.06), which may be interpreted as being practically significant (ES = 0.41). Likewise, the free carnitine also appeared to be lower in the fatigue group, as suggested by the proposed relation between these variables by the factor analysis (Biofactor 4). Finally, the proposed relation between uric acid and creatinine (Biofactor 3) was not statistically informative (FC = +1.04; p = 0.97 and FC = –1.05; p *=* 0.76), although biochemically relevant.

## Discussion

In this cohort study, we used two qualitative instruments (MSQ and PFS) and quantitative data (biochemical measurements) on 576 women found to suffer from periodic or chronic fatigue with no definitive tests or findings to establish a clinical diagnosis. Although the revised PFS that we used [36] was designed as a measure of fatigue in women with breast cancer, the scale was previously reported to be successfully used for different population groups [41][42][43] and the results presented here indicate that the revised PFS also provide a measure of fatigue. The state of fatigue in the patient group appeared to be highly diverse, but could be divided into five well-defined categories based on the fatigue scores from low to high. The structure of the measurements appeared to be represented by a bi-factor model (designated Energy Fatigue and Mental Fatigue), for which the reliability derived from the statistical analyses of the data proved to be high (Cronbach’s Alpha of 0.974 and 0.971 for the Energy and Mental Fatigue factors, respectively). This provides evidence that the four subscales of the PFS may represent distinct aspects of the fatigue experience as reported by patients with the condition, supported by the different trends in the biotransformation profiles observed for the Mainly Energy Fatigue and the Mainly Mental Fatigue groups (Table 1). A comparison of the clinical and biochemical data indicates that the canonical correlation between two sets of clinical variables was high (Cronbach’s Alpha = 0.789 for 15 main items of the MSQ) with smaller correlations with the original biochemical and factor data. Nevertheless, using CHAID analysis as the classification instrument of data derived from all three sets (MSQ, PFS and biochemical) provides a clear visual representation showing the advantage of the data from the MSQ used as a classification instrument and for detection of interaction between the three classes of variables (Fig 4). The observation that the 20 biochemical variables grouped into 8 biofactors, based on a PCA of the transformed data, emphasizes a strong underlying relationship among the biochemical variables which we developed as the biotransformation instrument and show that biotransformation and associated energy-derived parameters are indicative of undefined chronic fatigue observed in the patient group.

However, some limitations by focusing primarily on the results of statistical analysis need to be taken into account. Firstly, there is compelling evidence that the genes for glucuronidase (EC 3.2.1.31), sulphotransferase (EC 2.8.2.1) and glutathione-S-transferase (EC 2.5.1.18) are highly polymorphic. Likewise single nucleotide polymorphisms (SNPs) and copy number variations (CNVs) have been extensively reported for phase II genes. Each of these genetic aberrations affects the individuals’ Phase II biotransformation status in general, and may specifically be implicated in the acetoaminophen challenge used here, but this highly complex field was taken into account in the current manuscript. By contrast, we have previously reported on the genetic variation for the gene coding for glycine-N-acyl transferase (E.C. 2.3.1.13), involved in biotransformation of acetylsalicylic acid. Our main previous observations in this regard, are summarized in Table 2.

**Table 2:** Selected studies on the association between biotransformation of xenobiotics and genetic polymorphisms in the *GLYAT* gene.

PUBLICATION*	POLYMORPHIC VARIANT(S) AND RELEVANT INFORMATION ABOUT GLYCINE N-ACYLTRANSFERASE (GLYAT, EC 2.3.1.13)	OBSERVATION / INTERPRETATION
Badenhorst et al.[44]	A review paper in which: 1. It is argued that the major role of glycine conjugation is to dispose of the end products of phenylpropionate metabolism. 2. Genetic variation in the human GLYAT gene can influence the enzyme activity of a bacterially expressed recombinant human GLYAT. 3 The effects of six SNPs on enzyme indicated that the variant with the highest enzyme activity had the highest allele frequency, and that those with negative effects on enzyme activity occur very rarely. 4. The primary purpose of glycine conjugation is the detoxification of gut microbial benzoate and benzoyl-CoA, as illustrated through a schematic representation of the glycine conjugation pathway.	1. In humans the glycine N-acyltransferase enzyme (GLYAT) is important for biotransformation of xenobiotics which have a carboxylic acid group 2. Benzoate is potentially much more toxic than initially anticipated.
Van der Sluis et al.[45]	Site-directed mutagenesis was used to generate six non-synonymous SNP variants of the enzyme (K16N; S17T; R131H; N156S; F168L; R199C). The enzyme activities of the K16N, S17T and R131H variants were similar to that of the wild-type, whereas the N156S variant was more active, the F168L variant less active, and the R199C variant was inactive. An E227Q mutant, which lacks the catalytic residue proposed by Badenhorst et al.[46] was also synthesized.	1. The finding that SNP variations in the human GLYAT gene influence the kinetic properties of the enzyme may explain some of the inter-individual variation in glycine conjugation capacity.
Van der Sluis et al.[47]	With the exception of the South African Afrikaner Caucasian population (S156–86.9%; L61S156–10.7%), only two haplotypes namely S156 and T17S156, were identified in all populations as having the highest haplo- type frequencies (70% and 20% respectively).	1. This supports a previous suggestion that the S156 haplotype should be considered as the wild-type sequence. 2. The results in this study indicate that the GLYAT-ORF is highly conserved.
Nortje et al.[48]	A challenge test was done in the presence of two single nucleotide polymorphisms (SNPs) of the GLYAT gene with the highest allele frequencies across available global population data, namely theN156S (rs675815) and S17T(rs10896818).	1. All individuals (n = 10) were homozygous in this polymorphic region. 2. Haplotypes which may have a negative impact on enzyme activity were present at very low frequencies.

*- All of these listed publications came from our research group which has published a number of papers on glycine N-acyltransferase

Although these polymorphisms may contribute to inter-individual variation, the challenge tests incorporated variation coming from the availability of conjugation substrate (glycine), Coenzyme A sequestration and Adenosine Triphosphate (ATP) for xenobiotic activation to the corresponding CoA-derivative.

Measuring the effect of known polymorphisms on Phase II biotransformation falls beyond the scope of this research but warrants further investigation.

In our review [44] we argued that the major role of glycine conjugation is disposal of the end products of the gut microbiome, like those derived from polyphenols and phenylpropionate metabolism. Natural substrates for glycine conjugation would thus include benzoate, 4-hydroxybenzoate, 3-hydroxybenzoate, 4-aminobenzoate, and salicylate. Glucuronidation would not be ideal for the detoxification of these substances, although glucuronidated salicylate conjugates were observed in urine following aspirin dosing [49]. These substances were not detected in our analysis. However, significant inter-individual variation does exist in glycine conjugation capacity. To investigate the influence of SNPs in the GLYAT coding sequence on enzyme activity, we expressed and characterized a recombinant human GLYAT and introduced site-directed mutagenesis to generate six non-synonymous SNP variants of the enzyme [45]. The enzyme activities of the three variants were similar to that of the wild-type, one variant was more active, one less active, and one was inactive. Although these findings suggest that SNP variations in the human GLYAT gene may influence the enzymatic kinetic properties to account for inter-individual variation, our subsequent study [47]indicated that the GLYAT open reading frame (ORF) from a cohort of 1537 South African Afrikaner Caucasians appeared to be highly conserved, supporting the hypothesis that the glycine conjugation pathway is an essential detoxification pathway. It thus seems that genetic variability in the GLYAT gene did not compromise the observed biotransformation profiles following the salicylate intervention. Finally, we quantified the urinary excretion of p-aminohippuric acid over a period of ten hours in ten human volunteers following a p-aminobenzoic acid (PABA) challenge test[48]. All of the participants were able to metabolize PABA to such an extent that the product profile showed no further excretion of PAHA after 10 h, although considerable time dependent inter-individuality occurred. It thus appears that variations in the amounts of PABA conjugated to glycine may be attributed to factors like absorption (Phase 0), metabolism (Phase II) and excretion (Phase III), each phase with its own limiting factors which were call for a study in its own right for elucidation. Not-with-standing these limitations, the present study show how the analysis and integration of a wide-range of parameters can lead to the selection of both known and new indicators of biotansformation. Three observations emerged from the fatigue and the biochemical measurements in our patient group.

First, both the original and the transformed data indicate no significant difference between low and high fatigue groups for phase I transformation (Table 1 and Fig 5A and 5B). The ability to clear caffeine following the challenge is an indicator of the detoxification capacity of the hepatic Phase I pathway and is a recognized method to assess liver function and especially Phase I biotransformation via the CYP1A2 isoenzyme [50][51]. CYP1A2 contributes to the inactivation of several common drugs and dietary constituents, including acetaminophen[52]. The administration of a combination of challenge substances as used here, exclude measurement of different Phase I cytochrome P450 enzyme reactions simultaneously. However, Phase 1 biotransformation chemically modifies exogenous substances (caffeine, acetoaminophen and salicylic acid as used here) through the inducible CYP450 mixed function oxidase system, rendering them with improved ability for excretion (caffeine derivatives) or for phase II transformation catalysed by conjugation enzymes (glucuronidase, glutathione-S-transferase and sulphotransferase). The preferred detoxification modes in our patient group operated, however, through phase II glucuronidation (Fig 5C: the acetoaminophen challenge) and phase II glycination (Fig 5F: the salicylic acid challenge). Glucuronidation is a fairly common mode of detoxification, given the abundance of required co-factors and ubiquitous nature of these transferases [53], whereas our recent kinetic study on human GLYAT indicated that glycination is observed over a range of substrate concentrations as well as over a lengthy span of time to facilitates optimal detoxification [54]. The kinetic study also supports the observation of elevated excretion of salicyluric acid, the glycine conjugate of salicylic acid, in the high fatigue group, which points to an upregulated GLYAT enzyme system and subsequent greater usage of ATP for substrate activation [41]. Taken together, we propose that these findings suggest a possible causal relationship between high exposure to some xenobiotic substance(s) or environmental condition, upregulated and chronic phase II detoxification, ATP consumption and fatigue.

Second, differences in the profiles of 2,3-DHBA and ROS appear to implicate oxidative stress in patients who experience fatigue strongly with energy factors (the Mainly Energy Fatigue group), whereas patients more inclined to mental fatigue (the Mainly Mental Fatigue group) appeared to possess better antioxidant protection (FRAP and GSHt). Although mitochondrial dysfunction is recognized as the immediate cause of CFS symptoms like severe fatigue, a direct link to oxidative stress remains elusive [55], although increased oxidative stress remains a regularly observed condition in fatigue patients.

Third, the statistical metrics of biotransformation parameters for idiomatic fatigue, as shown here, do not comply with the requirements of diagnostic biomarkers [56] as defined for monogenetic or clinically well-defined diseases (e.g. significance [low (<0.001) p values], measures of sensitivity and specificity with high confidence intervals and validation outcomes). These biotransformation profiles underline the unique phenotypic/genetic response of individual humans towards endogenous or exogenous stressors, like toxicants. Moreover, depending on their chemical nature, some toxicants tend to bioaccumulate in the body and become stored in tissues or remain in serum [57], which most likely generates a polygenetic response in these individuals. These perturbed health profiles suggest the basis for a personalized approach to the treatment of fatigue patients, phrased as a “pragmatic clinical management” [58] in subjects with toxicant-related conditions, such as general and persistent fatigue.

The nuclear factor, erythroid 2-related factor 2-antioxidant response elements (Nrf2-ARE) as well as the androstane receptor (CAR) and pregnane receptor (PXR) are activated by a variety of endogenous and exogenous ligands[59][60] and are major determinants of phase II gene induction, so that upregulated phase II enzymes [61] may be promising targets for the treatment owing to their potent ability to detoxify harmful compounds and to combat ROS and oxidative stress. However, there is a dearth of clinical trials on inducers of Nrf2 and biotransformation through phase II involvement, which may be attributed to several concerns that preclude their translation to the clinic [62]. Nevertheless, an individualized approach implicating the application of biotransformation has been proposed by Sears and Genius [58], which we here extend, based on our observations made over several years of involvement with fatigue and biotransformation:

(a) Perform a comprehensive history to reasonably identify past and present xenobiotic exposures, while including clinical information similar to the present MSQ and PFS;

(b) apply the present broad-based biochemical instrument as a first approximation on the biotransformation status of the individual case;

(c) define a clinical approach to institute personal health-related treatment strategies directed towards the individual case.

The approach proposed here is very similar to that suggested by Beger et al. [63], in which a metabolite profile, clinical and lifestyle data were combined in a predictive patient profile, resulting in better patient stratification which facilitates personalized treatment and minimize the risk of the traditional treatment-failure approach. This precision medicine approach is fast gaining ground and has been described in a number of recent publications [64][65][66][67][68]. Incorporation of genomic data is part of our research unit’s future planning involving integration of genetic and metabolic profiles. According to Contrepois et al. [69], this approach and metadata (such as nutritional background, exercise and medical records) will allow the detection of early signs of aberrant biotransformation and will result in nutrition/lifestyle recommendations and efficient drug treatments and complements. Finally, our results have a direct bearing on the recent alert by the World Health Organization (WHO) regarding chronic non-communicable diseases. We believe that our contribution could be an important step towards unraveling a very complex syndromic phenomenon.

## Supporting information

S1 File (DOCX)
