# Supplementary material for: Biotransformation profiles from a cohort of chronic fatigue women in response to a hepatic detoxification challenge
Source: PLoS One. 2019 May 10;14(5):e0216298. doi: 10.1371/journal.pone.0216298 (PMC6510445; doi:10.1371/journal.pone.0216298)
Supplement: S1 File — (DOCX) [file pone.0216298.s001.docx]

**Supporting Information:**

**Biotransformation profiles from a cohort of chronic fatigue women in response to a hepatic detoxification challenge**

***Elardus Erasmus^1^, Francois E. Steffens^2^, Mari van Reenen^1^, B. Chris Vorster^1^, Carolus J. Reinecke^1^**

^1^ Human Metabolomics, North-West University (Potchefstroom Campus), Private Bag X6001, Potchefstroom 2522, South Africa

^2^ Department of Consumer Science, University of Pretoria, Pretoria 0002, South Africa

*: Corresponding author: Elardus Erasmus Lardus.Erasmus@nwu.ac.za 0027-18-299-2305

Concise and informative title: **Biotransformation and chronic fatigue**

Content:

1. Patient Selection:

Female patients who were complaining about fatigue were selected during visits to clinicians. Selection was based mainly on fatigue assessment by the clinicians and the completion of a demographic profile, the revised version of the Piper Fatigue Scale and the Medical Symptoms questionnaire. The questionnaires were part of an information guide about the challenge tests.

1. Informed consent:

Potential participating patients were also required to complete a section on informed consent which in essence contains information about the background and aim of the detoxification and oxidative stress evaluation, risks and possible discomforts, confidentiality, withdrawal, acknowledgement of explanation of the test procedure and finally the declaration of consent.

1. Statistics:

Supplementary table 1 reports the correlations coefficients (r) and associated p-values, based on Spearman’s rho, between suspected confounders (age, height, weight and BMI) and variables used to group cases (MSQ Overall, PFS Energy and PFS Mental scores) as well as biochemistry variables. Confounders were confirmed if showing a practically significant correlation (|r|≥0.5). While p-values are reported the level of significance was not considered as this metric is known to be affected by sample size. A sample size of 576, as used here, is likely to produce statistically significant effects of little practical importance. Given this criteria, no confounding effects could be detected. Note the non-parametric approach was used as some concerns about normality persisted, but more powerful parametric Pearson’s correlations were also performed. Pearson’s results were comparable and the same conclusions could be drawn.

**1) Patient Selection:**

1. **Informed Consent**

**Continue on next page …**

1. **Statistics:**

**Table A. – Correlation analysis with potential confounding effects.** The table reports the Spearman’s Rho non-parametric correlation coefficients (r) and associated significance levels (p-value) between variables pairs indicated as row and column headers. Correlation coefficients marked with a single asterisk (*) indicate significance at a 5% level, while correlation coefficients marked with a double asterisk (**) indicate significance at a 1% level.

|  | Spearman's Rho Correlation r (p-value) | | | |
| --- | --- | --- | --- | --- |
|  | Weight | Height | BMI | Age |
| MSQ | .087* | -0.01194 | .102* | 0.018704 |
|  | (0.038) | (0.775) | (0.014) | (0.654) |
| PFS_Energy | .100* | 0.021687 | .104* | -0.02876 |
|  | (0.016) | (0.604) | (0.013) | (0.491) |
| PFS_Mental | 0.041353 | -0.01059 | 0.048559 | -0.04974 |
|  | (0.322) | (0.8) | (0.245) | (0.233) |
| Creatinine | .109** | .118** | 0.064068 | -.391** |
|  | (0.009) | (0.005) | (0.125) | (<0.0001) |
| Uric acid | .115** | 0.067687 | .095* | -.229** |
|  | (0.006) | (0.105) | (0.023) | (<0.0001) |
| U Cratio | 0.011711 | -0.06503 | 0.046541 | .268** |
|  | (0.779) | (0.12) | (0.265) | (<0.0001) |
| Phase1 | -.190** | -0.05849 | -.180** | -0.03502 |
|  | (<0.0001) | (0.162) | (<0.0001) | (0.401) |
| Gluc | -0.04875 | -0.02834 | -0.03723 | -.172** |
|  | (0.243) | (0.498) | (0.372) | (<0.0001) |
| Sulph | -.130** | -0.02934 | -.125** | -0.03816 |
|  | (0.002) | (0.483) | (0.003) | (0.361) |
| Mercap | 0.012653 | 0.034901 | -0.0063 | -.100* |
|  | (0.762) | (0.404) | (0.88) | (0.016) |
| SAU | 0.005995 | 0.020809 | 0.008096 | -.121** |
|  | (0.886) | (0.619) | (0.846) | (0.004) |
| CatecholuM | -.091* | -0.05782 | -0.0605 | .120** |
|  | (0.028) | (0.167) | (0.147) | (0.004) |
| DHBAuM2_3 | -.229** | -.146** | -.166** | 0.02523 |
|  | (<0.0001) | (<0.0001) | (<0.0001) | (0.546) |
| DHBAuM2_5 | -.222** | -.149** | -.176** | -0.01479 |
|  | (<0.0001) | (<0.0001) | (<0.0001) | (0.723) |
| ROS | .153** | -0.03165 | .172** | -.124** |
|  | (<0.0001) | (0.449) | (<0.0001) | (0.003) |
| FRAP | -0.02681 | -0.02766 | -0.02214 | -0.03182 |
|  | (0.521) | (0.508) | (0.596) | (0.446) |
| GSHt | 0.042577 | .110** | 0.006483 | -0.05794 |
|  | (0.308) | (0.008) | (0.877) | (0.165) |
| TotalFreeC | .119** | 0.004537 | .120** | .156** |
|  | (0.004) | (0.914) | (0.004) | (<0.0001) |
| TotalAcylC | 0.027041 | -0.05035 | 0.047406 | 0.061815 |
|  | (0.517) | (0.228) | (0.256) | (0.138) |
| RatioTACTFC | -.164** | -.096* | -.126** | -.170** |
|  | (<0.0001) | (0.022) | (0.003) | (<0.0001) |
| Phase1Sulfation | -.127** | -0.05512 | -.111** | -0.00755 |
|  | (0.002) | (0.187) | (0.008) | (0.857) |
| Phase1Glycination | -.167** | -0.06549 | -.163** | 0.030533 |
|  | (<0.0001) | (0.117) | (<0.0001) | (0.465) |
| Phase1Glucuronidation | -.142** | -0.06849 | -.120** | 0.04645 |
|  | (0.001) | (0.101) | (0.004) | (0.266) |

Table A reports the correlation coefficients (r) and associated p-values, based on Spearman’s rho, between suspected confounders (age, height, weight and BMI) and variables used to group cases (MSQ Overall, PFS Energy and PFS Mental scores) as well as biochemistry variables. Confounders were confirmed if showing a practically significant correlation (|r|≥0.5). While p-values are reported the level of significance was not considered as this metric is known to be affected by sample size. A sample size of 576, as used here, is likely to produce statistically significant effects of little practical importance. Given this criteria, no confounding effects could be detected. Note the non-parametric approach was used as some concerns about normality persisted, but more powerful parametric Pearson’s correlations were also performed. Pearson’s results were comparable and the same conclusions could be drawn.
